# Association between copy number variation of complement component *C4 *and Graves' disease

**DOI:** 10.1186/1423-0127-18-71

**Published:** 2011-09-26

**Authors:** Yu-Huei Liu, Lei Wan, Chwen-Tzuei Chang, Wen-Ling Liao, Wen-Chi Chen, Yuhsin Tsai, Chang-Hai Tsai, Fuu-Jen Tsai

**Affiliations:** 1 Department of Medical Genetics and Medical Research, China Medical University Hospital, Taichung, Taiwan; 2 Graduate Institute of Integrated Medicine, China Medical University, Taichung, Taiwan; 3 School of Chinese Medicine, China Medical University, Taichung, Taiwan; 4 Department of Health and Nutrition Biotechnology, Asia University, Taichung, Taiwan; 5 Division of Endocrinology and Metabolism, Department of Medicine, China Medical University Hospital, Taichung, Taiwan; 6 Department of Endocrinology and Metabolism, College of Chinese Medicine, China Medical University, Taichung, Taiwan; 7 Department of Pediatrics, China Medical University Hospital, Taichung, Taiwan; 8 Department of Biotechnology, Asia University, Taichung, Taiwan; 9 School of Post-Baccalaureate Chinese Medicine, China Medical University, Taichung, Taiwan; 10 Department of Biotechnology, Asia University, Taichung, Taiwan; 11 Department of Biotechnology and Bioinformatics, Asia University, Taichung, Taiwan

## Abstract

**Background:**

Gene copy number of complement component *C4*, which varies among individuals, may determine the intrinsic strength of the classical complement pathway. Presuming a major role of complement as an effecter in peptide-mediated inflammation and phagocytosis, we hypothesized that *C4 *genetic diversity may partially explain the development of Graves' disease (GD) and the variation in its outcomes.

**Methods:**

A case-control study including 624 patients with GD and 160 healthy individuals were enrolled. CNV of *C4 *isotypes (*C4A *and *C4B*) genes were performed by quantitative real-time polymerase chain reaction analysis. Statistical comparison and identification of CNV of total *C4, C4 *isotypes (*C4A *and *C4B*) and *C4 *polymorphisms were estimated according to the occurrence of GD and its associated clinical features.

**Results:**

Individuals with 4, 2, and 2 copies of *C4*, *C4A *and *C4B *genes, especially those with A2B2 polymorphism may associate with the development of GD (p = 0.001, OR = 10.994, 95% CI: 6.277-19.255; p = 0.008, OR = 1.732, 95% CI: 1.190-2.520; p = 2.420 × 10-5, OR = 2.621, 95% CI: 1.791-3.835; and *p *= 1.395 × 10^-4^, OR = 2.671, 95% CI: 1.761-4.052, respectively). Although the distribution of copy number for total *C4*, *C4 *isotypes as well as *C4 *polymorphisms did not associate with the occurrence of goiter, nodular hyperplasia, GO and myxedema, <2 copies of *C4A *may associate with high risk toward vitiligo in patients with GD (*p *= 0.001, OR = 5.579, 95% CI: 1.659-18.763).

**Conclusions:**

These results may be further estimated for its clinical application on GD and the vitiligo in patients with GD.

## Background

Graves' disease (GD) is an organ-specific autoimmune thyroid disease [1]. It has been known that multiple factors, including the host's genetic factors as well as environmental factors, contribute to the etiology and severity of GD [2,3]. However, other forms of variation that might affect gene expression should also be considered.

A new paradigm in human genetics is high frequencies of interindividual variation in the copy number (CN) of specific genomic DNA segments. Copy number variation (CNV) loci often contain genes engaged in host-environment interactions, including those involved in immune functions, which results in susceptibility or resistance to autoimmune diseases [4-7], however, no significant association has been found between CNV and GD [6].

*Complement component C4 *(*C4*), located on chromosome 6q21.3, is encoded by 2 separate loci in the major histocompatibility complex class III region and derives 2 functionally distinct *C4A *and *C4B *isoforms [8]. The complement system is the main element of innate immunity and is regarded as the first line of defense against intrinsic and extrinsic antigens, leading to peptide-mediated inflammation, opsonization leading phagocytosis, the direct lysis of antigens [9]. Presuming a major role of complement as an effecter in peptide-mediated inflammation and phagocytosis, we hypothesized that *C4 *genetic diversity may partially explain the development of GD as well as the variation in its outcomes. Here we investigated the polymorphic variants of *C4 *that correlate with predisposition to this disease.

## Methods

### Patients and healthy individuals

A total of 624 patients (227 with GO and 397 without GO) with a confirmed diagnosis of GD and an appropriate control group with 160 healthy volunteers from China Medical University Hospital in Taiwan were enrolled and followed actively. All individuals provided informed consent as approved by the ethics committee of China Medical University Hospital. For the patients, diagnosis of GD and GO was followed the criteria set previously [10]. Full medical record abstraction was conducted to obtain demographics (age and gender); treatment and clinical features are summarized in Table 1. For the healthy individuals, those with matched for gender according to the female predominance of GD including 32 male (20.0%) and 128 female (80.0%). Age was different in healthy (27.4 ± 6.4 years) as compared to the patients with GD (41.1 ± 12.9 years) (p = 1.96 × 10^-34^).

**Table 1:** Background and demographic characteristics of patients with Graves' disease

Patients' characteristics	Healthy (160)	GD (624)	Myxedema		P-value	GO		P-value	Vitiligo		P-value
								
			No	Yes		No	Yes		No	Yes	
**Age at diagnosis**											
≤ 40	145 (90.6)	307 (49.2)	247 (47.0)	59 (60.2)	0.017	182 (45.8)	125 (55.1)	0.027	239 (46.9)	68 (59.6)	0.014
> 40	15 (9.4)	317 (50.8)	278 (53.0)	39 (39.8)		215 (54.2)	102 (44.9)		271 (53.1)	46 (40.4)	
**Gender**											
Male	32 (20.0)	133 (21.3)	110 (21.0)	22 (22.4)	0.739	74 (18.6)	59 (26.0)	0.031	107 (21.0)	26 (22.8)	0.700
Female	128 (80.0)	491 (78.7)	415 (79.0)	76 (77.6)		323 (81.4)	168 (74.0)		403 (79.0)	88 (77.2)	
**Treatment**											
Radioiodine											
No		601 (96.3)	504 (96.0)	96 (98.0)	0.345	389 (98.0)	212 (93.4)	0.003	489 (95.9)	112 (98.2)	0.226
Yes		23 (3.7)	21 (4.0)	2 (2.0)		8 (2.0)	15 (6.6)		21 (4.1)	2 (1.8)	
Thyroid gland surgery											
No		564 (90.4)	472 (89.9)	91 (92.9)	0.363	363 (91.4)	201 (88.5)	0.239	457 (89.6)	107 (93.9)	0.164
Yes		60 (9.6)	53 (10.1)	7 (7.1)		34 (8.6)	26 (11.5)		53 (10.4)	7 (6.1)	
**Clinical features**											
Goiter											
Grade 1-3		146 (23.5)	119 (22.8)	27 (27.6)	0.309	101 (25.5)	46 (20.4)	0.154	117 (23.1)	30 (26.3)	0.462
Grade 4-5		474 (76.5)	403 (77.2)	71 (72.4)		295 (74.5)	179 (79.6)		390 (76.9)	84 (73.7)	
Nodular hyperplasia											
No		483 (77.5)	434 (82.7)	49 (50.5)	2.880 × 10^-12^	301 (75.8)	182 (80.5)	0.175	430 (84.3)	53 (46.9)	6.670 × 10^-18^
Yes		140 (22.5)	91 (17.3)	49 (49.5)		96 (24.2)	44 (19.5)		80 (15.7)	60 (53.1)	
Myxedema											
No		525 (74.3)				305 (76.8)	220 (97.3)	1.35 × 10^-11^	507 (99.4)	18 (15.9)	8.900 × 10^-8^
Yes		98 (25.7)					92 (23.2)	6 (2.7)		3 (0.6)	95 (84.1)
Graves' ophthalmopathy											
No		397 (63.6)	305 (58.0)	92 (93.9)	1.350 × 10^-11^				295 (57.8)	102 (89.5)	2.200 × 10^-10^
Yes		227 (36.4)	220 (41.9)	6 (6.1)					215 (42.2)	12 (10.5)	
Vitiligo											
No		510 (81.7)	507 (96.6)	3 (3.1)	8.900 × 10^-8^	295 (74.3)	215 (94.7)	2.204 × 10^-10^			
Yes		114 (18.3)	18 (3.4)	95 (96.9)		102 (25.7)	12 (5.3)				

Abbreviations: GD, Graves, disease; GO, Graves' ophthalmopathy; SD, standard deviation; N, number.

### Genomic DNA extraction and quantification gene dosage of *C4A *and *C4B*

Genomic DNA was extracted from peripheral blood following the manufactory's suggestions (Qiagen). *C4 *gene dosage was assessed by quantitative real-time TaqMan^® ^PCR analysis (Applied Biosystems) as described in the previously published protocols with some modification [11]. Real-time PCR analysis was performed in 96-well optical plates on a 7900HT real-time PCR system (Applied Biosystems). Primers and probes specific for *C4A*, and *C4B *(common *C4A *and *C4B *forward primer "C4F": 5'-GCA GGA GAC ATC TAA CTG GCT TCT-3'; common *C4A *and *C4B *reverse primer "C4R": 5'-CCG CAC CTG CAT GCT CCT-3'; probe "*C4A*": FAM-ACC CCT GTC CAG TGT TAG; probe "*C4B*": FAM-ACC TCT CTC CAG TGA TAC. TaqMan^® ^Universal PCR Master Mix, No AmpErase^® ^uracil-DNA glycosylase (ABI catalog number 4326614), VIC-conjugated TaqMan^® ^*RNase P *control reagents (ABI catalog number 4316844), 250 nM of the respective FAM-conjugated TaqMan^® ^probes (*C4A *or *C4B*), the particular primers (300 nM *C4A *or *C4B*) in distilled water was contained in each of the distinct PCR batches. Appropriately prediluted genomic DNA (threshold cycle [C_T_] values for *RNase P *between 24 and 30) was added before start. CN of each target gene in each sample was determined from three separated experiments. Thermal cycler conditions were adjusted as follows: initial denaturation step for 10 minutes at 95°C; 40 cycles including denaturation for 15 seconds at 95 °C; and annealing/extension for 1 minute for 60°C. The data were analyzed using SDS 2.3 software (Applied Biosystems).

The C_T _value of *RNase P*, *C4A *or *C4B *was converted into a raw gene dosage by the formula nRAW_*C4X *_= 2 ^(CT*RNase P*)-(CT*C4X*)+1^, where *C4X *referred to *C4A *or *C4B *. Raw gene dosages of positive controls selected from the reference panel were plotted versus the actual gene dosages, and the resulting calibration curve served for determination of the actual copy number of unknown samples of this particular run.

### Statistical analysis

Statistical analysis was performed using the statistical package PASW for Windows (version 18.0; SPSS Inc.). The demographics of patients and healthy individuals were analyzed by the chi-square analysis. For those with 2 × 2 contingency tables, differences in the incidence of individuals with *C4 *gene CNs above and below the median or *C4A-C4B *polymorphisms between patients with or without indicated feature were evaluated using Fisher's exact test. For those above 2 × 2 contingency tables, differences in the incidence of individuals with *C4 *gene CNs above and below the median or *C4A-C4B *polymorphisms between patients with or without indicated feature were evaluated using Fisher's exact test, and the two-tailed *p *value was estimated by 100,000 Monte Carlo simulations with 99% confidence intervals (CI) (99% confidence for the simulation result). Odds ratios (ORs) and 95% CIs were estimated from logistic regression models adjusting for confounding variables as shown in Table 1.

## Results

### CNV of *C4 *genes is associated with susceptibility to GD

The distribution of copy number for total *C4*, *C4 *isotypes as well as *C4 *polymorphisms according to the presence of GD is shown in Table 2. No individuals had a full deficiency of *C4 *alleles. After adjusting for age, individuals with 4 copies of *C4 *gene were more susceptible to GD (*p *= 0.001, OR = 10.994, 95% CI: 6.277-19.255) as compared to those without, whereas those with <4 copies of *C4 *gene tended to prevent from GD (*p *= 0.003, OR = 0.512, 95% CI: 0.338-0.776) as compared to those without. The distribution of *C4A *and *C4B *among individuals with or without GD was further investigated. For *C4A *gene, individuals with 2 copies of *C4A *increased the risk toward GD (*p *= 0.008, OR = 1.732, 95% CI: 1.190-2.520) whereas those with <2 copies of *C4A *reduced the risk toward GD (*p *= 0.01, OR = 0.584, 95% CI: 0.360-0.948). For *C4B *gene, individuals with 2 copies of *C4B *increased the risk toward GD (*p *= 2.420 × 10^-5^, OR = 2.621, 95% CI: 1.791-3.835) whereas those without 2 copies of *C4B *reduced the risk toward GD (*p *= 0.008, OR = 0.487, 95% CI: 0.322-0.738 for those with <2 copies *C4B*; *p *= 0.015, OR = 0.545, 95% CI: 0.347-0.856 for those with >2 copies *C4B *respectively). Polymorphism analysis indicated tat individuals with the most common polymorphism (37.3%), A2B2, with 2.671-fold risk toward GD (*p *= 1.395 × 10^-4^, OR = 2.671, 95% CI: 1.761-4.052) as compared to those without. These results indicate that individuals with 4, 2 and 2 copies of *C4*, *C4A *and *C4B *genes, especially those with A2B2 polymorphism may have higher risk, whereas those with<4, <2 and ≠2 copies of *C4*, *C4A *and *C4B *genes may have lower risk toward GD, respectively.

**Table 2:** Distribution of *C4 *polymorphisms in individuals with or without Graves' disease

Variations	GD		***P *value, individual**^**a**^ **[OR (95%CI), individual]**^**c**^	***P *value **^**b**^	**OR (95%CI)**^**d**^
	No, N (%)	Yes, N (%)			
***C4 *CNV**					
4	57 (35.6)	314 (50.3)	0.001 [10.994 (6.277-19.255)]	0.002	(Reference)
< 4	52 (32.5)	134 (21.5)	0.003 [0.512 (0.338-0.776)]		0.389 (0.245-0.615)
> 4	51 (31.9)	176 (28.2)	0.361		0.497 (0.317-0.780)
***C4A *CNV**					
2	83 (51.9)	395 (63.3)	0.008 [1.732 (1.190-2.520)]	0.011	(Reference)
< 2	33 (20.6)	79 (12.7)	0.010 [0.584 (0.360-0.948)]		0.509 (0.307-0.843)
> 2	44 (27.5)	150 (24.0)	0.365		0.628 (0.404-0.977)
***C4B *CNV**					
2	67 (41.9)	377 (60.4)	2.420 × 10^-5 ^[2.621 (1.791-3.835)]	1.328 × 10^-4^	(Reference)
< 2	53 (33.1)	143 (22.9)	0.008 [0.487 (0.322-0.738)]		0.374 (0.240-0.584)
> 2	40 (25)	104 (16.7)	0.015 [0.545 (0.347-0.856)]		0.391 (0.241-0.636)
***C4 *polymorphisms**					
A2B2	39 (24.4)	254 (40.7)	1.395 × 10^-4 ^[2.671 (1.761-4.052)]	3.87 × 10^-6^	(Reference)
A2B1	22 (13.8)	78 (12.5)	0.672		0.409 (0.219-0.763)
A3B2	16 (10.0)	64 (10.3)	0.924		0.539 (0.273-1.064)
A2B3	5 (3.1)	44 (7.1)	0.067		0.961 (0.343-2.697)
A3B1	10 (6.3)	32 (5.1)	0.574		0.373 (0.159-0.876)
A1B2	6 (3.8)	34 (5.4)	0.384		0.687 (0.257-1.836)
Other	62 (38.8)	118 (18.9)			0.242 (0.148-0.396)

Abbreviations: GD, Graves' disease; CNV, copy number variation; OR, odds ratio; CI, confidence interval; N, number.

^a^Individual *C4 *CNVs and polymorphisms between individuals with or without GD were evaluated by Fisher's exact test using 2 × 2 contingency tables.

^b ^CNV of *C4, C4A and C4B *between individuals with or without GD were evaluated by Fisher's exact test using 3 × 2 contingency tables.*C4 *polymorphisms between individuals with or without GD were evaluated by Fisher's exact test using 7 × 2 contingency tables. The *p *value was estimated by 100,000 Monte Carlo simulations with 99% confidence intervals (CI).

^c^ORs and 95% CIs were estimated from logistic regression models adjusting for age.

^d^ORs and 95% CIs were estimated from logistic regression models adjusting for age.

### CNV of *C4 *genes did not significantly associated with myxedema and GO

We also estimated the association between polymorphism of *C4 *genes and clinical features of GD. CNV of *C4 *genes showed association with susceptibility toward GO, vitiligo and myxedema, but not goiter or nodular hyperplasia as estimated by Fisher's exert test (data not shown). After adjusting for age, nodular hyperplasia, GO, and vitiligo, the distribution of copy number for total *C4*, *C4 *isotypes as well as *C4 *polymorphisms did not associate with the occurrence of myxedema (Table 3).

**Table 3:** Distribution of *C4 *polymorphisms in Graves' disease patients with or without myxedema

Variations	Myxedema		***P *value, individual**^**a**^ **[OR (95%CI), individual]**^**c**^	***P *value **^**b**^	**OR (95%CI)**^**d**^
	No, N (%)	Yes, N (%)			
***C4 *CNV**					
4	265 (50.5)	48 (49.0)	0.826		(Reference)
< 4	100 (19.0)	34 (34.7)	0.001 [1.884 (0.538-6.597)]	4.900 × 10^-4^	1.714 (0.447-6.575)
> 4	160 (30.5)	16 (16.3)	0.005 [0.617 (0.166-2.289)]		0.761 (0.186-3.122)
***C4A *CNV**					
2	336 (64.0)	58 (59.2)	0.364		(Reference)
< 2	57 (10.9)	22 (22.5)	0.003 [0.627 (0.164-2.404)]	0.008	0.511 (0.122-2.134)
> 2	132 (25.1)	18 (18.4)	0.159		0.496 (0.117-2.106)
***C4B *CNV**					
2	317 (60.4)	59 (60.2)	1		(Reference)
< 2	115 (21.9)	28 (28.6)	0.152	0.168	1.163 (0.298-4.542)
> 2	93 (17.7)	11 (11.2)	0.072		0.552 (0.125-2.443)
***C4 *polymorphisms**					
A2B2	217 (41.3)	36 (36.7)	0.434	0.050	(Reference)
A2B1	63 (12.0)	15 (15.3)	0.405		1.895 (0.307-11.710)
A3B2	58 (11.0)	6 (6.1)	0.202		1.371 (0.163-11.522)
A2B3	41 (7.8)	3 (3.1)	0.130		0.558 (0.029-10.789)
A3B1	26 (5.0)	6 (6.1)	0.619		0.333 (0.032-3.499)
A1B2	23 (4.4)	11 (11.2)	0.013 [1.094 (0.137-8.709)]		1.009 (0.103-9.841)
Other	97 (18.5)	21 (21.4)			0.735 (0.163-3.310)

Abbreviations: GD, Graves' disease; GO, Graves' ophthalmopathy; CNV, copy number variation; OR, odds ratio; CI, confidence interval; N, number.

^a^Individual *C4 *CNVs and polymorphisms between GD patients with or without myxedema were evaluated by Fisher's exact test using 2 × 2 contingency tables.

^b ^CNV of *C4, C4A and C4B *between GD patients with or without myxedema were evaluated by Fisher's exact test using 3 × 2 contingency tables.*C4 *polymorphisms between GD patients with or without myxedema were evaluated by Fisher's exact test using 7 × 2 contingency tables. The *p *value was estimated by 100,000 Monte Carlo simulations with 99% confidence intervals (CI).

^c^ORs and 95% CIs were estimated from logistic regression models adjusting for age, nodular hyperplasia, GO and vitiligo.

^d^ORs and 95% CIs were estimated from logistic regression models adjusting for age, nodular hyperplasia, GO and vitiligo.

The distribution of copy number for total *C4*, *C4 *isotypes as well as *C4 *polymorphisms according to the presence of GO is shown in Table 4. The relationship between *C4 *CNV status and GO was not significant (*p *= 0.396). The distribution of *C4A *and *C4B *among GD patients with and without GO were further investigated. After adjusting for age, gender, radioiodine treatment, vitiligo and myxedema, neither isotypes nor polymorphisms of *C4 *was significantly associated with GO, although GD patients with <2 copies (0 or 1) of the *C4A *gene were less susceptible to GO (*p *= 0.014, OR = 0.549, 95% CI: 0.303-0.998) as compared to those with 2 copies of *C4A*, and those with A3B1 polymorphism were less susceptible to GO (*p *= 0.001, OR = 0.374, 95% CI: 0.146-0.960) as compared to those with A2B2 polymorphism. These results indicate that neither isotypes nor polymorphisms of *C4 *was significantly associated with GO, however, as compared to GD patients with 2 copies of *C4A *or those with A2B2 polymorphism, those with <2 copies of *C4A *or those with A3B1 might be protected against the development of GO, respectively.

**Table 4:** Distribution of *C4 *polymorphisms in Graves' disease patients with or without Graves' ophthalmopathy

Variations	GO		***P *value, individual**^**a**^ **[OR (95%CI), individual]**^**c**^	***P *value **^**b**^	**OR (95%CI)**^**d**^
	No, N (%)	Yes, N (%)			
***C4 *CNV**					
4	196 (49.4)	118 (52.0)	0.561	0.396	(Reference)
< 4	92 (23.2)	42 (18.5)	0.188		0.978(0.614-1.558)
> 4	109 (27.5)	67 (29.5)	0.581		1.029(0.687-1.540)
***C4A *CNV**					
2	238 (39.9)	157 (69.2)	0.025 [1.436 (0.994-2.075)]	0.014	(Reference)
< 2	61 (15.4)	18 (7.9)	0.008 [0.590 (0.328-1.059)]		0.549 (0.303-0.998)
> 2	98 (24.7)	52 (22.9)	0.628		0.772 (0.509-1.169)
***C4B *CNV**					
2	229 (57.7)	148 (65.2)	0.074	0.186	(Reference)
< 2	97 (24.4)	46 (20.3)	0.276		0.806 (0.520-1.249)
> 2	71 (17.9)	33 (14.5)	0.316		0.697 (0.430-1.132)
***C4 *polymorphisms**					
A2B2	149 (37.5)	105 (46.3)	0.035 [1.283 (0.900-1.828)]	0.005	(Reference)
A2B1	53 (13.4)	25 (11.0)	0.451		0.796 (0.449-1.411)
A3B2	37 (9.3)	27 (11.9)	0.338		1.067 (0.596-1.912)
A2B3	29 (7.3)	15 (6.6)	0.871		0.734 (0.366-1.476)
A3B1	25 (6.3)	7 (3.1)	0.091		0.374 (0.146-0.960)
A1B2	28 (7.1)	6 (2.6)	0.026 [0.451(0.176-1.160)]		0.374 (0.153-1.056)
Other	65 (16.4)	40 (17.6)			0.894 (0.549-1.455)

Abbreviations: GD, Graves' disease; GO, Graves' ophthalmopathy; CNV, copy number variation; OR, odds ratio; CI, confidence interval; N, number.

^a^Individual *C4 *CNVs and polymorphisms between GD patients with or without GO were evaluated by Fisher's exact test using 2 × 2 contingency tables.

^b ^CNV of *C4, C4A and C4B *between GD patients with or without GO were evaluated by Fisher's exact test using 3 × 2 contingency tables.*C4 *polymorphisms between GD patients with or without GO were evaluated by Fisher's exact test using 7 × 2 contingency tables. The *p *value was estimated by 100,000 Monte Carlo simulations with 99% confidence intervals (CI).

^c^ORs and 95% CIs were estimated from logistic regression models adjusting for age, gender, ever received radioiodine treatment, myxedema and vitiligo.

^d^ORs and 95% CIs were estimated from logistic regression models adjusting for age, gender, ever received radioiodine treatment, myxedema and vitiligo.

### GD patients with <2 copies of *C4A *had higher risk toward vitiligo

The distribution of copy number for total *C4*, *C4 *isotypes as well as *C4 *polymorphisms according to the presence of vitiligo is shown in Table 4. After adjusting with age, nodular hyperplasia, GO and myxedema, patients with <2 copies of *C4A *had a 5.153-fold increased risk of vitiligo (*p *= 2.650 × 10^-4^, OR = 5.153, 95% CI: 1.629-16.300). It remained significant even when compared with GD patients with 2 copies of *C4A *(*p *= 0.001, OR = 5.579, 95% CI: 1.659-18.763, Table 5). These results indicate that <2 copies of *C4A *may increase the risk for vitiligo in patients with GD.

**Table 5:** Distribution of *C4 *polymorphisms in Graves' disease patients with or without vitiligo

Variations	Vitiligo		***P *value, individual**^**a**^ **[OR (95%CI), individual]**^**c**^	***P *value **^**b**^	**OR (95%CI)**^**d**^
	No, N (%)	Yes, N (%)			
***C4 *CNV**					
4	258 (50.6)	56 (49.1)	0.836	0.002	(Reference)
< 4	97 (19.0)	37 (32.5)	0.002 [1.297 (0.434-3.874)]		1.334 (0.415-4.289)
> 4	155 (30.4)	21 (18.4)	0.011 [0.987 (0.362-2.691)]		1.076 (0.370-3.133)
***C4A *CNV**					
2	330 (64.7)	65 (57.0)	0.133		(Reference)
< 2	52 (10.2)	27 (23.7)	2.650 × 10^-4 ^[5.153 (1.629-16.3000]	0.001	5.579 (1.659-18.763)
> 2	128 (25.1)	22 (19.3)	0.225		1.289 (0.414-4.013)
***C4B *CNV**					
2	310 (60.8)	67 (58.8)	0.751		(Reference)
< 2	112 (22.0)	31 (27.2)	0.267	0.414	1.133 (0.355-3.614)
> 2	88 (17.3)	16 (14.0)	0.487		2.107 (0.687-6.467)
***C4 *polymorphisms**					
A2B2	213 (41.8)	41 (36.0)	0.292	0.03	(Reference)
A2B1	62 (12.2)	16 (14.0)	0.638		0.889 (0.175-4.524)
A3B2	57 (11.2)	7 (6.1)	0.125		0.756 (0.132-4.335)
A2B3	40 (7.8)	4 (3.5)	0.154		1.111 (0.151-8.205)
A3B1	25 (4.9)	7 (6.1)	0.638		1.484 (0.199-11.089)
A1B2	22 (4.3)	12 (10.5)	0.019 [2.035(0.335-12.368)]		2.745 (0.384-19.599)
Other	91 (17.8)	27 (23.7)			3.471 (1.046-11.525)

Abbreviations: GD, Graves' disease; GO, Graves' ophthalmopathy; CNV, copy number variation; OR, odds ratio; CI, confidence interval; N, number.

^a^Individual *C4 *CNVs and polymorphisms between GD patients with or without vitiligo were evaluated by Fisher's exact test using 2 × 2 contingency tables.

^b ^CNV of *C4, C4A and C4B *between GD patients with or without vitiligo were evaluated by Fisher's exact test using 3 × 2 contingency tables.*C4 *polymorphisms between GD patients with or without vitiligo were evaluated by Fisher's exact test using 7 × 2 contingency tables. The *p *value was estimated by 100,000 Monte Carlo simulations with 99% confidence intervals (CI).

^c^ORs and 95% CIs were estimated from logistic regression models adjusting for age, nodular hyperplasia, myxedema and GO.

^d^ORs and 95% CIs were estimated from logistic regression models adjusting for age, nodular hyperplasia, myxedema and GO.

## Discussion

Several functionally relevant single nucleotide polymorphisms are characteristic of GD and GO [12,13], but no relevant CNV has been reported [14]. In the present study, we found that the CNV of *C4*, *C4A *or *C4B *may associate with the development of GD. In addition, <2 copies of *C4A *may associate with development of vitiligo in patients with GD. To the best of our knowledge, this is the first study to report that the linkage among CNV of *C4 *genes, GD and GD-associated vitiligo. Our results provide new information which may be applied clinically.

C4 involves in the classical pathway which is triggered by interaction of the Fc portion of an antibody or C-reactive protein with C1q. It has been shown that the copy number of *C4*, *C4A *or *C4B *positively correlated with the protein levels of total C4, C4A or C4B, respectively [7]. In our results, individuals with 4, 2, and 2 copies of *C4*, *C4A *or *C4B *have higher risk whereas those with deficiencies of *C4*, *C4A *or *C4B *have lower risk toward GD. One possibility is that a deficiency of complement may lead to ineffective opsonization, lytic activity or impairment of B-cell memory, by which reduce tissue injury [15]. Unfortunately, the mechanisms by which *C4 *abnormality contributes to the protection of organ-specific autoimmunity are poorly understood. Nevertheless, whether a potential gene-gene or gene-environment interaction is involved in susceptibility to GD needs to be further investigated [16]. This study provides a substantial amount of data that may help to clarify the role of *C4 *genes in this disorder. It is only through investigations of diverse populations that researchers can expect to dissect the complex genetics involved. In addition, functional studies of susceptibility genes using appropriate animal models could allow for an assessment of their role in the disease process.

However, it may play a different regulatory role in systemic autoimmune diseases. Low level of C4 complements in sera has been found in several autoimmune diseases [17-21]. In addition, the presence of *C4A *null allele that results in partial C4 deficiency have shown to be risk factor for susceptibility in systemic lupus erythematosus (SLE) and the SLE-related renal damage [7,19]. A hypothesis is that complement may participate in the presentation of self-antigens to developing B cells by which protects against responses to self-antigens and subsequence promoting the elimination of self-reactive lymphocytes [9]. The pathogenesis of vitiligo, similar to SLE, is characterized by the destruction of cutaneous melanocytes which due to another antibody-induced hypopigmentation. Experiments in knockout mice have demonstrated that complement deficient can cause the destruction of pigment cells leading to vitiligo-like depigmentation [21]. Our results revealed that deficiency of *C4A *may enhance the development of vitiligo in GD patients, implying exist of an alternative pathway for the deficiency of complement.

What is interesting is that although we explored the relationship of *C4 *CNV to GD as well as other GD clinical features, only the lower copies of *C4A*, but not *C4B*, were associated with higher risk of vitiligo. Because it appears that C4A binds to amino group-containing antigens such as immune complex, whereas C4B binds to hydroxyl group-containing antigens such as bacteria, this result may provide another view to support the hypotheses that the pathogenesis of vitiligo may be more relevant to the existence of the immune complex than the pathogen. In addition, recent studies have identified that the risk locus within the major histocompatibility complex region on chromosome 6q may be associated with vitiligo in both Chinese Han population and American population [22,23]. It may be interesting to investigate the gene-gene interaction between *C4 *polymorphism and the vitiligo risky locus. Moreover, although confirmation of these results in larger samples is warranted, it would be interesting to further investigate the functional role of *C4A *in the development of vitiligo.

## Conclusion

This study provides evidence that the CNV of *C4*, *C4A *or *C4B *may associate with the development of GD and <2 copies of *C4A *may associate with development of vitiligo in patients with GD. These results may be further estimated for its application on predicting the occurrence of GD and the clinical outcome in patients with GD which might aid in the diagnosis of the disease and the development of therapeutic strategies.

## List of abbreviations

(GD): Graves' disease; (GO): Graves' ophthalmopathy; (CNV): copy number variation; (CN): copy number; (SLE): systemic lupus erythematosus.

## Competing interests

The authors declare that they have no competing interests.

## Authors' contributions

YHL designed the study, managed the literature searches, undertook the statistical analysis, and wrote the draft of the manuscript. LW designed and performed the experiments. CTC and WCC recruited and maintained the clinical information of participants. LWLL and TYT undertook the statistical analysis. CHT and FJT directed the study and reviewed the results. All authors contributed to and have approved the final manuscript.

## References

[B1] Mishra A Mishra SK Multicentre study of thyroid nodules in patients with Graves' disease (Br J Surg 2000; 87: 1111-13) Br J Surg 2001 88 2 313 11167890 10.1046/j.1365-2168.2001.01729-3.x

[B2] Tomer Y Huber A The etiology of autoimmune thyroid disease: a story of genes and environment J Autoimmun 2009 32 3-4 231 239 10.1016/j.jaut.2009.02.007 19307103 PMC3561494

[B3] McGrogan A Seaman HE Wright JW de Vries CS The incidence of autoimmune thyroid disease: a systematic review of the literature Clin Endocrinol (Oxf) 2008 69 5 687 696 10.1111/j.1365-2265.2008.03338.x 18673466

[B4] Fanciulli M Petretto E Aitman TJ Gene copy number variation and common human disease Clin Genet 77 3 201 213 10.1111/j.1399-0004.2009.01342.x 20002459

[B5] Schaschl H Aitman TJ Vyse TJ Copy number variation in the human genome and its implication in autoimmunity Clin Exp Immunol 2009 156 1 12 16 10.1111/j.1365-2249.2008.03865.x 19220326 PMC2673736

[B6] Fanciulli M Norsworthy PJ Petretto E Dong R Harper L Kamesh L Heward JM Gough SCL de Smith A Blakemore AIF Owen CJ Pearce SHS Teixeira L Guillevin L Graham DSC Pusey CD Cook HT Vyse TJ Aitman TJ FCGR3B copy number variation is associated with susceptibility to systemic, but not organ-specific, autoimmunity Nature Genetics 2007 39 6 721 723 10.1038/ng2046 17529978 PMC2742197

[B7] Yang Y Chung EK Wu YL Savelli SL Nagaraja HN Zhou B Hebert M Jones KN Shu Y Kitzmiller K Blanchong CA McBride KL Higgins GC Rennebohm RM Rice RR Hackshaw KV Roubey RA Grossman JM Tsao BP Birmingham DJ Rovin BH Hebert LA Yu CY Gene copy-number variation and associated polymorphisms of complement component C4 in human systemic lupus erythematosus (SLE): low copy number is a risk factor for and high copy number is a protective factor against SLE susceptibility in European Americans Am J Hum Genet 2007 80 6 1037 1054 10.1086/518257 17503323 PMC1867093

[B8] Yu CY Whitacre CC Sex, MHC and complement C4 in autoimmune diseases Trends Immunol 2004 25 12 694 699 10.1016/j.it.2004.10.006 15530841

[B9] Carroll MC The role of complement and complement receptors in induction and regulation of immunity Annu Rev Immunol 1998 16 545 568 10.1146/annurev.immunol.16.1.545 9597141

[B10] Liu YH Chen RH Chen WC Tsai Y Wan L Tsai FJ Disease association of the CD103 polymorphisms in Taiwan Chinese Graves' ophthalmopathy patients Ophthalmology 117 8 1645 1651 10.1016/j.ophtha.2009.12.037 20417566

[B11] Szilagyi A Blasko B Szilassy D Fust G Sasvari-Szekely M Ronai Z Real-time PCR quantification of human complement C4A and C4B genes BMC Genet 2006 7 1 16403222 10.1186/1471-2156-7-1 PMC1360677

[B12] Zeitlin AA Simmonds MJ Gough SC Genetic developments in autoimmune thyroid disease: an evolutionary process Clin Endocrinol (Oxf) 2008 68 5 671 682 10.1111/j.1365-2265.2007.03075.x 18081880

[B13] Jacobson EM Tomer Y The genetic basis of thyroid autoimmunity Thyroid 2007 17 10 949 961 10.1089/thy.2007.0153 17824829

[B14] Fanciulli M Norsworthy PJ Petretto E Dong R Harper L Kamesh L Heward JM Gough SC de Smith A Blakemore AI Froguel P Owen CJ Pearce SH Teixeira L Guillevin L Graham DS Pusey CD Cook HT Vyse TJ Aitman TJ FCGR3B copy number variation is associated with susceptibility to systemic, but not organ-specific, autoimmunity Nat Genet 2007 39 6 721 723 10.1038/ng2046 17529978 PMC2742197

[B15] Markiewski MM Lambris JD The role of complement in inflammatory diseases from behind the scenes into the spotlight Am J Pathol 2007 171 3 715 727 10.2353/ajpath.2007.070166 17640961 PMC1959484

[B16] Davies EJ Steers G Ollier WE Grennan DM Cooper RG Hay EM Hillarby MC Relative contributions of HLA-DQA and complement C4A loci in determining susceptibility to systemic lupus erythematosus Br J Rheumatol 1995 34 3 221 225 10.1093/rheumatology/34.3.221 7728395

[B17] Lachmann PJ Complement deficiency and the pathogenesis of autoimmune immune complex disease Chem Immunol 1990 49 245 263 2140942

[B18] Beurskens FJ van Dijk H Robins DM Does complement component C4A protect from autoimmune disease? Immunol Today 1997 18 4 199 9136457 10.1016/s0167-5699(97)84667-1

[B19] Seelen MA Daha MR The role of complement in autoimmune renal disease Autoimmunity 2006 39 5 411 415 10.1080/08916930600739688 16923541

[B20] Chen M Daha MR Kallenberg CG The complement system in systemic autoimmune disease J Autoimmun 34 3 J276 286 10.1016/j.jaut.2009.11.014 20005073

[B21] Trcka J Moroi Y Clynes RA Goldberg SM Bergtold A Perales MA Ma M Ferrone CR Carroll MC Ravetch JV Houghton AN Redundant and alternative roles for activating Fc receptors and complement in an antibody-dependent model of autoimmune vitiligo Immunity 2002 16 6 861 868 10.1016/S1074-7613(02)00327-8 12121667

[B22] Jin Y Birlea SA Fain PR Gowan K Riccardi SL Holland PJ Bennett DC Herbstman DM Wallace MR McCormack WT Kemp EH Gawkrodger DJ Weetman AP Picardo M Leone G Taieb A Jouary T Ezzedine K van Geel N Lambert J Overbeck A Spritz RA Genome-Wide Analysis Identifies a Quantitative Trait Locus in the MHC Class II Region Associated with Generalized Vitiligo Age of Onset J Invest Dermatol 2011 Jun;131 6 1308 12 Epub 2011 Feb 17 21326295 10.1038/jid.2011.12 PMC3172680

[B23] Quan C Ren YQ Xiang LH Sun LD Xu AE Gao XH Chen HD Pu XM Wu RN Liang CZ Li JB Gao TW Zhang JZ Wang XL Wang J Yang RY Liang L Yu JB Zuo XB Zhang SQ Zhang SM Chen G Zheng XD Li P Zhu J Li YW Wei XD Hong WS Ye Y Zhang Y Wu WS Cheng H Dong PL Hu DY Li Y Li M Zhang X Tang HY Tang XF Xu SX He SM Lv YM Shen M Jiang HQ Wang Y Li K Kang XJ Liu YQ Sun L Liu ZF Xie SQ Zhu CY Xu Q Gao JP Hu WL Ni C Pan TM Yao S He CF Liu YS Yu ZY Yin XY Zhang FY Yang S Zhou Y Zhang XJ Genome-wide association study for vitiligo identifies susceptibility loci at 6q27 and the MHC Nat Genet 42 7 614 618 10.1038/ng.603 20526339

